# Eating self for not be eaten: Pancreatic cancer suppresses self-immunogenicity by autophagy-mediated MHC-I degradation

**DOI:** 10.1038/s41392-020-0209-8

**Published:** 2020-06-12

**Authors:** Xing Huang, Xiaozhen Zhang, Xueli Bai, Tingbo Liang

**Affiliations:** 1 Zhejiang Provincial Key Laboratory of Pancreatic Disease, the First Affiliated Hospital, School of Medicine, Zhejiang University, Hangzhou, 310003 Zhejiang China; 2 Department of Hepatobiliary and Pancreatic Surgery, the First Affiliated Hospital, School of Medicine, Zhejiang University, Hangzhou, 310003 Zhejiang China

**Keywords:** Tumour immunology, Gastrointestinal cancer

In a recent study in *Nature*,^1^ Yamamoto et al. reported that MHC-I is a selective substrate of autophagy in tumor immune evasion. Furthermore, it was reported that autophagy-mediated lysosomal degradation of MHC-I suppresses pancreatic cancer immunogenicity and the effectiveness of immunotherapy. These findings indicate the potential of autophagy inhibition as a strategy to activate anti-pancreatic cancer immunity.^2^

In addition to targeted therapy, immunotherapy is regarded as a promising approach to cancer treatment and exhibits huge market potential. However, compared with other solid tumors, the progress of immune-related therapy for pancreatic cancer to date is extremely limited. In terms of the underlying cause, the current consensus is that, compared with other solid tumors, the microenvironment in pancreatic cancer is unique, including a physical barrier composed of cancer-associated fibroblasts and an extracellular matrix, as well as the immune barrier formed by tumor-associated macrophages, regulatory T cells, and myeloid-derived suppressor cells. It is precisely the existence of this dual physico-immune barrier that leads to the occurrence of a degree of immune evasion in pancreatic cancer, and further hinders the effectiveness of pancreatic cancer immunotherapy. However, tumor immunity is ultimately a confrontation between the tumor and the immune system (although the “battlefield” is represented by the tumor microenvironment), and thus, the mechanism underlying the immune evasion in pancreatic cancer is a continuing focus of research.

**Fig. 1:**
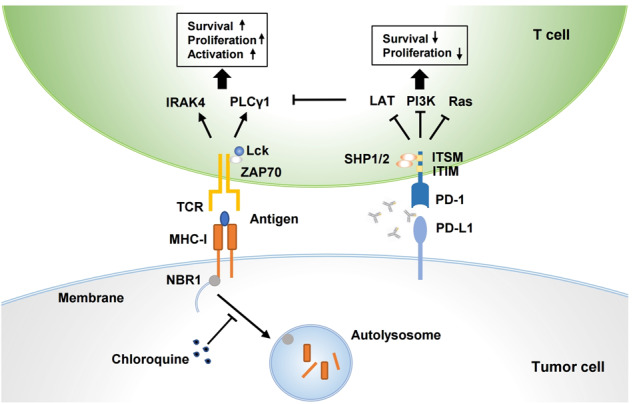
Autophagy inhibition potentiates anti-pancreatic cancer immunotherapy. Tumoral MHC-I contributes to TCR-mediated antigen recognition by T cells, while PD-1–PD-L1 interaction causes T cell dysfunction and immune evasion. MHC-I is degraded by autophagy in pancreatic cancer, and thus results in deficiency of antigen presentation and consequent ineffectiveness of immunotherapy. Combination of autophagy inhibitor and PD-1/PD-L1 blockade can improve therapeutic efficacy of pancreatic cancer, showing the potential to be a promising strategy for cancer therapy

Interestingly, a recent study by Yamamoto et al.^1^ revealed a role for dysregulated expression of major histocompatibility complex class I (MHC-I) molecule, an immunomodulatory protein expressed on cell surfaces, in the immune evasion of pancreatic cancer, an issue that has been drawn wide attention (Fig. 1). The amount of MHC-I on the surface of cancer cells determines the efficiency of antigen presentation as well as the strength of anti-tumor immune responses. In fact, it has been long ago observed that MHC-I content is much lower than normal levels, or completely lost in more than 60% of pancreatic tumors, although the mechanism of such downregulation is still unclear. Intriguingly, Yamamoto et al.^1^ observed that most MHC-I molecules in pancreatic cancer cells were present in autophagosomes and autolysosomes, rather than on the cell surface. Autophagy is an important intracellular degradation pathway that replenishes raw materials for cell growth by recycling discarded organelles and proteins to maintain cellular homeostasis. Yamamoto et al. found that pancreatic cancer cells restricted the quantity of MHC-I molecules on their surface via the autophagy pathway, thereby hindering their presentation of antigens. Further studies revealed that the autophagy-related receptor NBR1 binds MHC-I molecules on the surface of pancreatic cancer cells and targets them for transport into autophagosomes and autolysosomes, where they are decomposed by lysosomal proteases. Thus, it can be hypothesized that inhibition of autophagy by drugs such as chloroquine or genetic engineering would result in restoration of the surface expression levels of MHC-I molecules, thereby enhancing the presentation of antigens and anti-tumor T cell responses. Finally, in a mouse model of pancreatic cancer, Yamamoto et al.^1^ showed that autophagy inhibition led to increasing infiltration of cytotoxic T cells, reduced tumor growth, and also significantly enhanced the synergistic effects of PD-1 and CTLA-4 monoclonal antibodies. In addition, MHC-I was also found to be degraded by autophagy in non-small cell lung cancer, which highlights the potential of strategies targeting autophagy to enhance immune responses.

Notably, autophagy is not only involved in immune evasion in pancreatic cancer, but is also closely related to the resistance of pancreatic cancer to traditional therapies. Mutations in the KRAS gene, which is an important member of the RAS oncogenic family, have been observed in at least 90% of pancreatic cancer patients. KRAS-mediated aberrant activation of the MEK and ERK signaling pathways promotes abnormal cell differentiation and sustained growth, thereby driving the development and progression of pancreatic cancer.^3^ However, various drugs targeting KRAS mutations or its downstream signaling pathways do not improve therapeutic efficacy of pancreatic cancer in the clinic. In the recent issue of *Nature Medicine*,^4,5^ Kinsey et al. and Bryant et al. reported their almost simultaneous discovery that autophagy is significantly enhanced in pancreatic cancer cells by inhibiting key molecules in the KRAS-RAF-MEK-ERK signaling pathway. Mechanistically, inhibition of the KRAS signaling cascade impairs glycolysis and mitochondrial function in tumor cells, which enhances the dependence of tumor tissue growth on the products from autophagic degradation. In the meanwhile, inhibition of the KRAS signaling cascade also causes activation of AMPK signaling and inhibition of mTORC1 signaling, both of which are well-established key regulators of autophagy, leading to a direct increase of autophagy. Since the products of the catabolism associated with the autophagic process provide nutrients necessary for maintaining the normal metabolism of tumors, therapeutic approaches targeting this signaling pathway cannot significantly inhibit tumor cell growth. However, the autophagy inhibitor hydroxychloroquine combined with MEK or ERK inhibitors has been shown to provide a significant synergistic inhibitory effect on pancreatic cancer both at the in vitro cellular level and in vivo mouse models, even in clinical trials. Furthermore, the effect of this regimen is superior to that of the current standard for the treatment of pancreatic cancer (gemcitabine + albumin-bound paclitaxel). Moreover, this combination strategy is not limited to pancreatic cancer and shows similar antitumor effects in NRAS-mutated melanoma and BRAF-mutated colorectal cancer. Therefore, a combination regimen that simultaneously targets the abnormal KRAS signaling pathway and the autophagic process in cancer cells is implicated as a novel and feasible treatment modality for pancreatic cancer patients.

In conclusion, these studies provide a solid theoretical basis for the application of strategies targeting autophagy in pancreatic cancer immunotherapy, and has important significance in terms of clinical translation and social benefits. However, the regulation of autophagy in tumor development may change under different conditions, or even play a completely opposite role; therefore, combination therapy targeting autophagy and tumor immunity requires further exploration before this approach can be widely recommended. Furthermore, detailed evaluation of this novel combination regimen in clinical trials is needed to determine precisely which types of pancreatic cancer patients will benefit most from this kind of therapy.
